# Structural and Functional Determinants of ARIA-H Risk in Anti-Amyloid Monoclonal Antibodies: A Comparative Mechanistic Framework for Alzheimer's Immunotherapy Development

**DOI:** 10.2174/011570159X391766250806091602

**Published:** 2025-08-19

**Authors:** Dinghao An, Xinxin Zou, Yun Xu

**Affiliations:** 1 Department of Neurology, Nanjing Drum Tower Hospital, Chinese Academy of Medical Sciences & Peking Union Medical College, Nanjing, China;; 2 Department of Neurology, Nanjing Drum Tower Hospital, Affiliated Hospital of Medical School, Nanjing University, Nanjing, China;; 3 Jiangsu Key Laboratory for Molecular Medicine and Institute of Translational Medicine for Brain Critical Diseases, Nanjing University, Nanjing, China;; 4 Nanjing Neurology Clinical Medical Center, and Nanjing Gulou Hospital, Brain Disease and Brain Science Center, Nanjing, China

**Keywords:** Amyloid-related imaging abnormalities with hemorrhage, ARIA-H, Aβ binding, Alzheimer’s disease, monoclonal antibodies, neuropharmacology

## Abstract

**Introduction:**

Amyloid-beta-targeting monoclonal antibodies (mAbs) for Alzheimer's disease frequently induce amyloid-related imaging abnormalities with hemorrhage (ARIA-H), yet systematic comparisons of ARIA-H incidence across therapeutic agents remain limited. Post-approval research prioritizes dosing over mechanism, leaving unresolved whether ARIA-H variations originate from intrinsic mAb properties. We address two gaps: comparative ARIA-H risk stratification among clinically available/investigational mAbs, and elucidation of structural/functional features influencing ARIA-H susceptibility.

**Methods:**

A systematic comparison of seven mAbs (donanemab, aducanumab, bapineuzumab, lecanemab, gantenerumab, crenezumab, solanezumab) was conducted, analyzing clinical trial data and molecular characteristics.

**Results:**

ARIA-H incidence ranked as follows (highest to lowest): donanemab > aducanumab > bapineuzumab > lecanemab > gantenerumab > crenezumab > solanezumab. Five mAb-specific determinants emerged: (1) Types of Aβ Binding: Enhanced clearance of mature amyloid plaques correlated with elevated ARIA-H risk. (2) Polymer binding Affinity: Reduced small oligomer-binding capacity predicted higher ARIA-H incidence. (3) Epitope location: N-terminal-targeting mAbs showed greater ARIA-H incidence *vs*. mid/C-terminal binders. (4) Fc region structure: IgG4-based constructs showed higher ARIA-H incidence than IgG1 analogs. (5) Clearance kinetics: Rapid attainment of amyloid reduction thresholds amplified ARIA-H incidence.

**Discussion:**

We identify a risk hierarchy for ARIA-H among anti-Aβ mAbs and link specific mAb biophysical properties—Aβ binding type, affinity for soluble oligomers, epitope specificity, Fc structure, and plaque clearance dynamics—directly to ARIA-H pathogenesis.

**Conclusion:**

These findings establish a mechanistic framework for ARIA-H risk and provide concrete molecular predictors to guide antibody engineering strategies. Prioritizing mAbs with controlled amyloid clearance, C-terminal binding domains, and IgG1 frameworks may enhance therapeutic safety, advancing precision immunotherapy for Alzheimer's disease.

## 1 INTRODUCTION

The advent of amyloid-targeting monoclonal antibodies (mAbs) has reshaped early Alzheimer's disease (AD) treatment [1]. In September 2022, the Phase III Clarity AD clinical trial data for Leqembi showed that the drug reduced the rate of cognitive and functional decline in patients with early symptomatic AD by 27% over 18 months [2]. In July 2023, the Phase III TRAILBLAZER-ALZ 2 study of donanemab indicated that It slowed cognitive and functional decline by 35% over 18 months in some early Alzheimer’s patients. Both Leqembi, administered bi-weekly, and Donanemab, administered every four weeks, are effective in clearing Aβ plaques in the brain and significantly slowing the progression of early AD [3]. Anti-β-amyloid mAbs offer significant value in the early treatment of Alzheimer's disease. The FDA has approved three mAb drugs: Aducanumab, Lecanemab, and Donanemab [4]. However, Biogen announced that Aducanumab had been withdrawn from the market [5, 6]. They chose to reallocate resources toward other Alzheimer's treatments, particularly lecanemab (Leqembi), which has received traditional FDA approval and is considered more promising in terms of clinical benefit and market viability. While these agents effectively reduce amyloid burden, their clinical utility is limited by ARIA. All monoclonal antibodies inevitably cause ARIA, which includes two subtypes: (1) ARIA-E (Effusion or Edema), detected by FLAIR sequences as brain tissue swelling or cortical abnormalities; and (2) ARIA-H (Hemorrhage), detected by GRE/T2* or SWI sequences as new brain microbleeds, hemorrhage, and superficial siderosis [7, 8]. Most ARIA-E cases are asymptomatic, but some patients may experience headache, confusion, nausea, dizziness, seizures, and, in rare cases, death [9-11]. ARIA-H risk factors include age, use of antithrombotic medications, history of stroke, and cerebral amyloid angiopathy [12, 13]. Different mAbs have different incidence rates of ARIA-H [14]. Initially, Aducanumab has the highest ARIA-H occurrence rate to our knowledge [15]. Aβ exists in several forms, including Monomer (Individual Aβ molecules); Oligomers (Small aggregates of Aβ); which include: Dimers and Trimers (Aggregates of 2-3 molecules), Hexamers to Octamers (Aggregates of 6-8 molecules), Larger Oligomers (Aggregates of 8-12 molecules); Protofibrils (early, less organized aggregates of Aβ); Fibrils (Elongated, structured aggregates); Amyloid Plaques (Larger, insoluble deposits) [16, 17].

Monoclonal antibodies have been carefully administered to eligible patients [18]. However, ARIA-H can still occur, potentially impacting not only the treatment of AD but also the patient's overall prognosis [19]. The appearance of ARIA is undoubtedly closely related to the dosage and frequency of administration [20]. The pathophysiology of the ARIA-H is not yet fully understood. Some researchers suggest that it involves two interconnected mechanisms: (1) Aβ fragment-induced vascular inflammation triggered by rapid plaque clearance, and (2) destabilization of cerebral amyloid angiopathy (CAA)-compromised vessels. When mAbs bind vascular Aβ deposits, they generate pro-inflammatory cleavage products (*e.g*., Aβ1-16) that activate microglial secretion, degrading vascular basement membranes. This process is exacerbated in APOE ε4 carriers, where impaired Aβ clearance amplifies perivascular inflammation. In addition, emerging evidence suggests ARIA-H risk varies substantially across mAbs, correlating with pharmacological properties [21, 22]. However, given the different targets and characteristics of various monoclonal antibodies, it is essential not to attribute ARIA-H solely to dosage and administration frequency. It is crucial to conduct further relevant research on the monoclonal antibodies currently on the market and those undergoing large-scale randomized controlled trials.

Research on monoclonal antibodies targeting Aβ still faces certain limitations. First, regarding the incidence of ARIA-H, there are only a few retrospective analyses of individual mAbs, lacking comprehensive comparative studies. We aim to understand the occurrence of ARIA-H across different mAbs, either available on the market or under investigation. Second, after the approval of mAbs, current research has focused on dosing frequency and drug dosage. It remains unclear whether the occurrence rate of ARIA-H is related to the specific characteristics of mAbs themselves. The properties of various mAbs differ, and their mechanisms are also not uniform. The varying incidence of ARIA among different mAbs raises the question of whether ARIA-H is associated with the specific characteristics of the mAbs. This research addresses critical gaps in understanding ARIA-H determinants while proposing mechanistic links between mAb design and cerebrovascular toxicity, provoking new insights for optimizing next-generation immunotherapies.

## 2 MATERIALS AND METHODS

### 2.1 Study Design

We analyzed seven amyloid-beta-targeting monoclonal antibodies (mAbs) with completed Phase III trials: aducanumab, bapineuzumab, crenezumab, donanemab, gantenerumab, lecanemab, and solanezumab. Primary data were collated from two independent meta-analyses of Phase III clinical trials for these seven monoclonal antibodies [15, 23]. Data from Jeremic *et al*. [15] include the six monoclonal antibodies, while data from Qiao *et al*. [23] include four mAbs. The following data were extracted from each trial: authors, clinical trial number, country, sample size, gender, age, Alzheimer's disease severity, baseline Mini-Mental State Examination (MMSE) scores, ApoE allele carrier status, and incidence of ARIA-H to assess the safety profile of anti-amyloid beta monoclonal antibodies. For binary ARIA-H outcomes, results were expressed as log risk ratios (RR) with 95% confidence intervals (CI). When raw data were unavailable (*e.g*., missing standard deviations (SD) or reporting of only *p*-values/ranges), established estimation methods were applied to calculate SD and 95% CI from available parameters, as described by Wan *et al*. (2014) in BMC Medical Research Methodology [24]. The selection between random-effects and fixed-effects models depended on statistical heterogeneity values for each outcome. A random-effects model was employed when significant heterogeneity was present (I^2^ > 50%), whereas a fixed-effects model was applied for outcomes with low heterogeneity (I^2^ ≤ 50%). The data for Bapineuzumab are sourced from Qiao Y, and the data for the other six mAbs come from Jeremic D. As for Ponezumab: due to its failure in Phase II trials, Pfizer discontinued the development of this drug in 2016, and therefore, it is excluded from our study [25]. The data sources are detailed in Tables **S1** and **S2**.

For mechanistic variable selection, five mAb-specific features were evaluated: the form of monoclonal antibody binding to Aβ protein, polymer binding affinity, binding epitope, Fc subtype of the monoclonal antibody, and the Aβ clearance rate. ARIA-H incidence was modeled as a categorical dependent variable. The odds ratio (OR) was utilized for data assessment, with an OR not equal to 1 and a 95% confidence interval excluding 1 used as the criterion for statistical significance.

To ensure cross-comparability of ARIA-H incidence across seven mAbs, we implemented a three-stage standardization protocol. Firstly, we use Table **S2** data for bapineuzumab and aducanumab as baseline references; we calculated normalization coefficients through linear regression analysis (R^2^ >0.85) to harmonize these measurements with corresponding data in Table **S1**. Secondly, after applying these validated coefficients, we proportionally adjusted aducanumab and crenezumab data in Table **S1** to align with bapineuzumab's standardized framework, ensuring consistent quantification of amyloid clearance parameters across all agents. Thirdly, we converted these data to match those for Bapineuzumab and Aducanumab in Table **S1** and found a coefficient. Subsequently, we converted the data for Aducanumab and Crenezumab in Table **S1** to match the data for Bapineuzumab and Crenezumab in Table **S1** according to the ratio coefficients. All transformations followed CONSORT-derived principles for inter-trial data harmonization, with sensitivity analyses confirming coefficient robustness (95% CI overlap across bootstrap iterations).

### 2.2 Search Strategy and Eligibility Criteria

Original literature was sourced from searches on Google Scholar, PubMed, ClinicalTrials.gov, Embase, Cochrane Library, and Web of Science for published randomized controlled trials (RCTs) on anti-amyloid beta (Aβ) monoclonal antibody therapies for Alzheimer’s disease (AD). The following keyword combinations were used: “Alzheimer’s disease,” “sporadic,” “mild cognitive impairment,” “monoclonal antibody,” “passive immunotherapy,” “Aducanumab,” “BIIB037,” “Lecanemab,” “BAN-2401,” “Solanezumab,” “LY2062430,” “Bapineuzumab,” “AAB-001,” “Gantenerumab,” “Crenezumab,” and “donanemab.” Manual reviews of references from relevant meta-analyses, reviews, pooled analyses, and included trials were conducted, retaining only the most informative, largest sample published RCTs. Clinical trial report quality was assessed using the Jadad scale, which evaluates five criteria: randomization, blinding of participants and researchers, blinding of outcome assessments, withdrawal reporting, and statistical methods [26]. Studies included in the meta-analysis must: (1) be phase III, parallel-design, randomized, placebo-controlled trials; (2) have a Jadad score ≥3; (3) enroll participants with mild cognitive impairment (MCI) or sporadic AD at any stage; (4) have sufficient sample size (≥200 participants for cognitive and safety measures); and (5) be published/presented in any language. AD patients in primary studies could receive standard symptomatic AD medications. Exclusion criteria: (1) narrative/systematic reviews, meta-analyses, or phase I/II/IV trials; (2) Jadad score <3; (3) single-arm trials, prospective cohort studies, case-control studies, cross-sectional studies, case reports, opinions/editorials, or post-hoc/secondary analyses of primary studies; (4) studies involving familial AD or other dementias; (5) failure to report cognitive/functional outcomes; and (6) inclusion of <200 patients for cognitive and safety outcome analyses.

### 2.3 Data Analysis

#### 2.3.1 Comparative Risk Analysis

For pairwise comparisons of ARIA-H incidence between monoclonal antibodies (mAbs): OR <1: Higher risk in the first mAb listed. OR >1: Higher risk in the second mAb listed.

#### 2.3.2 Overall ARIA-H Risk Stratification

ARIA-H incidence across seven mAbs was standardized against placebo using ORs, with sensitivity analyses verifying robustness.

#### 2.3.3 Subgroup Analysis Framework

Five mechanistic variables were evaluated using a controlled variable approach. (Types of Aβ Binding, Polymer clearance Affinity, Epitope location, Fc region structure, Clearance kinetics) We controlled variables by this protocol: Four variables matched, and one variable with distinct properties between mAb pairs (Tables **S1-S2** and literature-based characterization). ORs relative to placebo computed for each subgroup.

#### 2.3.4 Subgroup 1: Types of Aβ Binding

• Monomer: Bapineuzumab, Crenezumab, Solanezumab.

• Oligomers: Aducanumab, Bapineuzumab, Crenezumab, Gantenerumab, Lecanemab.

• Protofibrils: Lecanemab.

• Fibrils: Aducanumab, Bapineuzumab, Crenezumab, Donanemab, Gantenerumab, Lecanemab.

• Plaques: Aducanumab, Donanemab, Gantenerumab, Lecanemab.

#### 2.3.5 Subgroup 2: Polymer Affinity

• 6-8 and 8-12 polymers: Gantenerumab≈Lecanemab.

• 2-3 polymers: Gantenerumab>Lecanemab.

#### 2.3.6 Subgroup 3: Binding Epitope

• N-terminal binders: Aducanumab, Bapineuzumab, Donanemab, Lecanemab.

• C-terminal binders: Solanezumab, Crenezumab.

• Dual binders: Gantenerumab.

#### 2.3.7 Subgroup 4: Fc Region Subtype

• IgG4: Crenezumab.

• IgG1: Donanemab, Lecanemab, Gantenerumab, Aducanumab, Bapineuzumab, Crenezumab, Solanezumab.

• 8. Subgroup 5: Aβ Clearance Rate.

• Donanemab > Lecanemab ≈ Gantenerumab > Aducanumab > Bapineuzumab > Crenezumab ≈ Solanezumab (Ranked by centiloid reduction rate).

## 3 RESULTS

### 3.1 ARIA-H Risk Stratification Across Anti-Aβ mAbs

After integration, the OR and 95% CI for ARIA-H occurrence in Phase III clinical trials of the 7 monoclonal antibodies compared to placebo are as follows: Aducanumab OR (95% CI)(4.44 (3.75, 5.28)), Bapineuzumab OR (95% CI) (2.51 (2.13, 2.93)), Crenezumab OR (95% CI) (1.21 (0.83, 1.76)), Donanemab OR (95% CI) (5.77 (3.39, 10.26)), Gantenerumab OR (95% CI) (1.81 (1.32, 2.49)), Lecanemab OR (95% CI) (2.12 (1.60, 2.83)), Solanezumab OR (95% CI) (1.15 (0.79, 1.68)).

Risk hierarchy: Donanemab > Aducanumab > Bapineuzumab > Lecanemab > Gantenerumab > Crenezumab > Solanezumab (For detailed information, Table **1**).

### 3.2 Mechanistic Drivers of ARIA-H Susceptibility

**Fig. (1):**
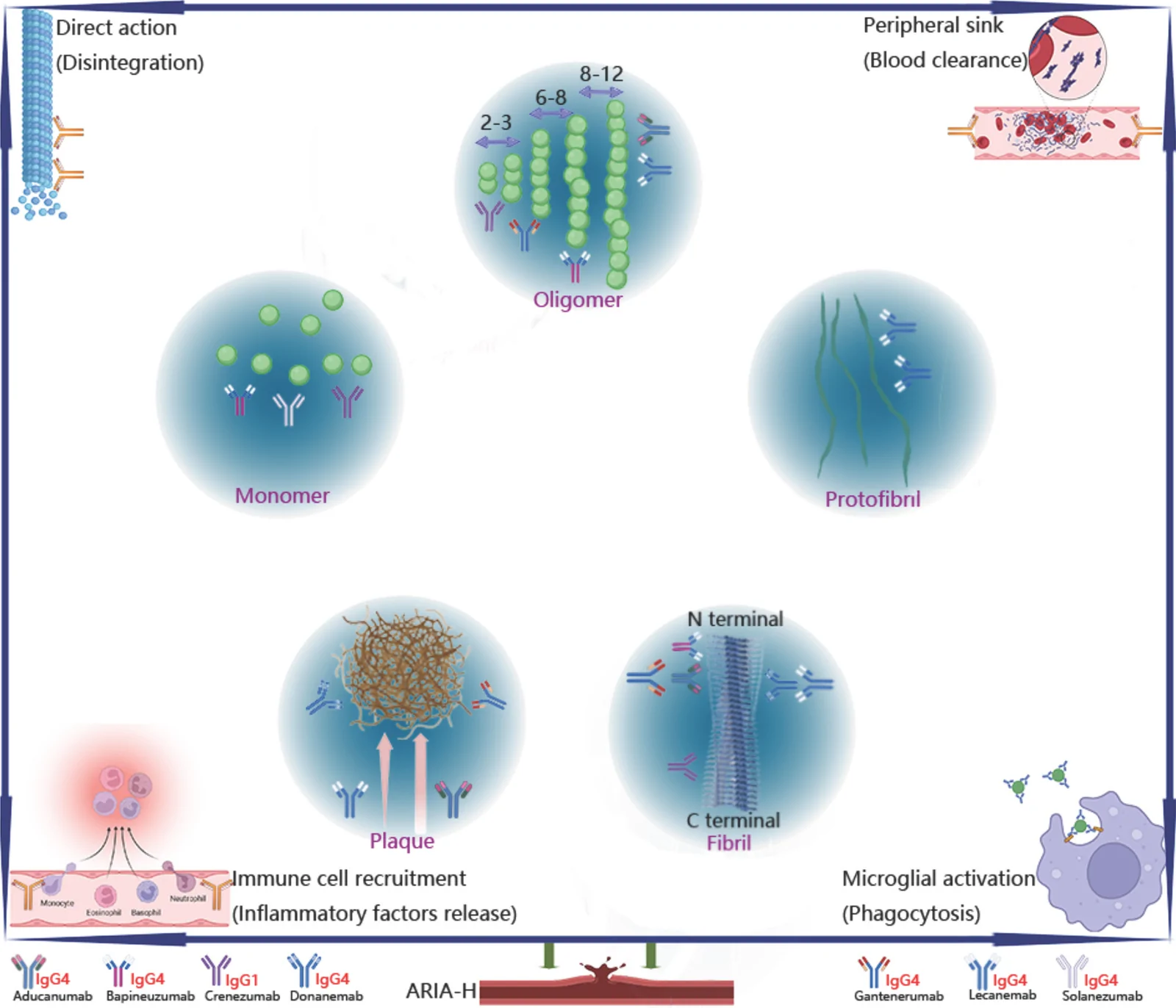
Monoclonal antibodies with different characteristics clear different Aβ. For different monoclonal antibodies, the four surrounding mechanisms are potential modes of action. Below are seven monoclonal antibodies, with five circles corresponding to five forms of Aβ. Based on the unique characteristics of each monoclonal antibody, the Aβ clearance rates are depicted in the five diagrams. In the Plaque circle, sharp arrows represent fast clearance, while blunt arrows indicate slow clearance for plaques.

**Fig. (2):**
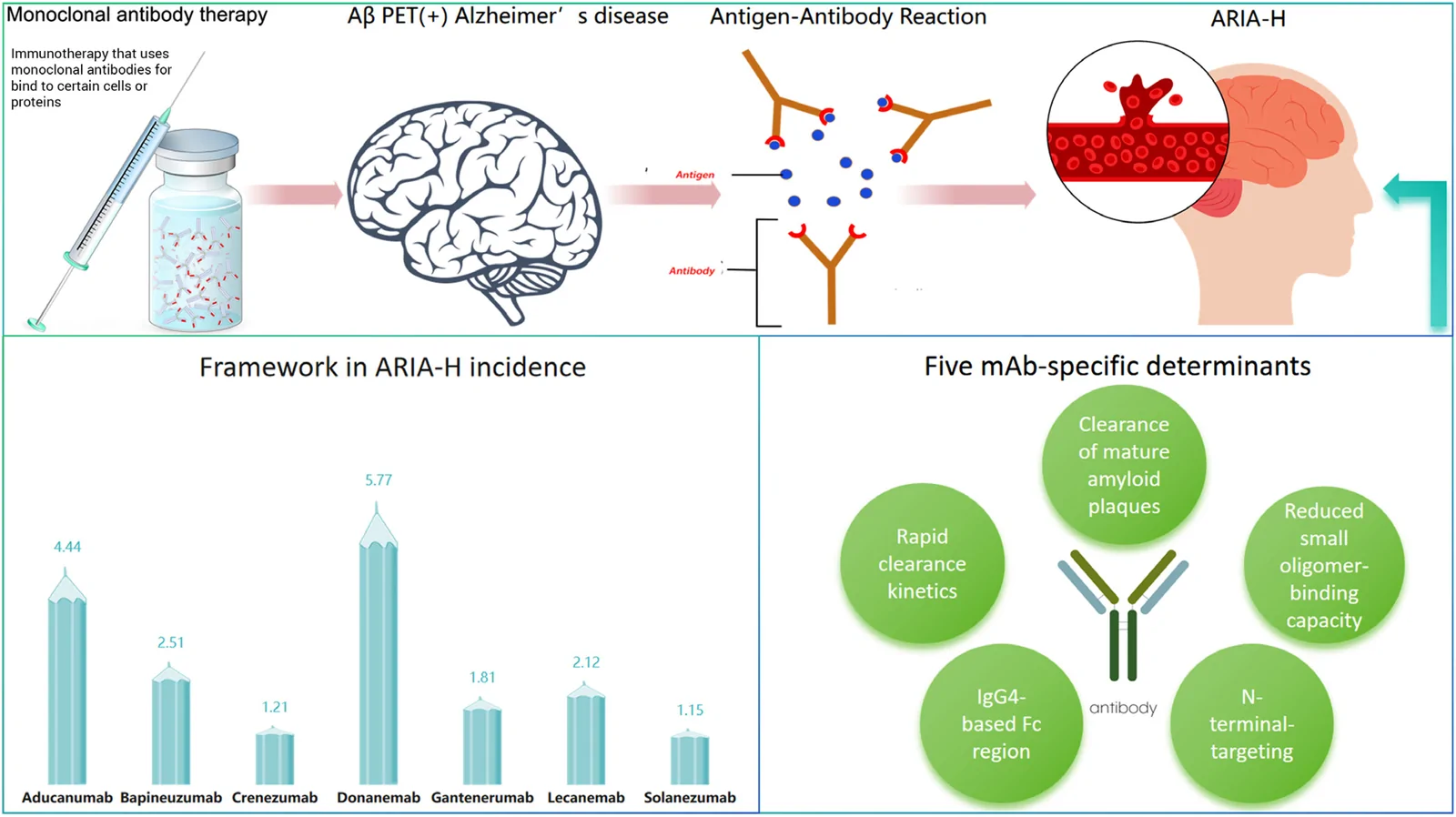
Mechanistic Framework Links Antibody Properties to ARIA-H Risk in Alzheimer's Immunotherapy. Upper panel: illustrates the potential occurrence of ARIA-H during the use of anti-Aβ monoclonal antibodies. Lower-Left Panel: Presents a vertical bar chart quantifying ARIA-H incidence rates (%) across seven clinically investigated mAbs, ranked hierarchically as follows (highest to lowest). Lower-Right Panel: Presents five modules to delineate critical mAb characteristics susceptible to ARIA-H.

The occurrence of ARIA-H is associated with the characteristics of Aβ monoclonal antibodies as follows: (Figs. **1** and **2**, Figs. **S1**, and **S2** for details).

#### 3.2.1 Subgroup 1: Aβ Binding Specificity

Comparing mAbs with distinct Aβ-targeting profiles: Solanezumab: highest efficacy against soluble Aβ monomers; Crenezumab: superior clearance of soluble oligomers; Lecanemab: most effectively eliminates insoluble protofibrils/fibrils; Aducanumab: greatest potency against mature amyloid plaques. We therefore conducted pairwise comparisons among these four agents to evaluate their differential effects on ARIA-H risk profiles.

Solanezumab *vs*. Crenezumab: OR (95% CI): 1.39 (0.82, 2.36); Solanezumab *vs*. Lecanemab: OR (95% CI): 2.43 (1.52, 3.92); Solanezumab *vs*. Aducanumab: OR (95% CI): 5.09 (3.37, 7.73); Crenezumab *vs*. Lecanemab: OR (95% CI): 0.57 (0.36, 0.91); Crenezumab *vs*. Aducanemab: OR (95% CI): 3.68 (2.43, 5.53); Lecanemab *vs*. Aducanumab: OR (95% CI): 2.1 (1.5, 2.92).

ARIA-H risk Hierarchy: Plaque/fibril-targeting > protofibril > oligomer > monomer (Aducanumab > Lecanemab > Crenezumab > Solanezumab).

#### 3.2.2 Subgroup 2: Polymer Binding Affinity

We conducted comparisons among Lecanemab and Gantenerumab on ARIA-H risk profiles, matched for similar Aβ targets, clearance rate, epitope, and IgG1 subtype.

• Lecanemab *vs*. Gantenerumab: OR (95% CI): 0.85 (0.56, 1.31).

• Enhanced small oligomer binding may reduce ARIA-H risk despite similar fibril engagement.

#### 3.2.3 Subgroup 3: Epitope Specificity

We conducted comparisons among Bapineuzumab and Crenezumab on ARIA-H risk profiles, matched for IgG1 subtype, similar oligomer/fibril targeting, and clearance rate.

• Crenezumab *vs*. Bapineuzumab: OR (95% CI): 2.07 (1.43,2.75).

• N-terminal engagement showed amplified ARIA-H risk.

#### 3.2.4 Subgroup 4: Fc Region Subtype

We conducted comparisons between Crenezumab and Solanezumab on ARIA-H risk profiles, matched for

C-terminal binding, similar Aβ monomer/oligomer targets, and clearance rate.

• Solanezumab *vs*. Crenezumab: OR (95% CI): 1.39 (0.82, 2.36).

• IgG4's reduced FcγR binding showed a non-significant amplified ARIA-H risk.

#### 3.2.5 Subgroup 5: Aβ Clearance Kinetics

We conducted comparisons among Aducanumab and Lecanemab on ARIA-H risk profiles, matched for N-terminal binding, IgG1 subtype, and fibril/plaque targeting.

• Lecanemab *vs*. Aducanumab: OR (95% CI): 2.1 (1.5, 2.92).

• Faster amyloid removal (Δcentiloids/month) showed amplified ARIA-H risk.

## 4 DISCUSSION

Our systematic analysis establishes five key determinants governing ARIA-H susceptibility in anti-Aβ monoclonal antibodies, resolving longstanding uncertainties about variability in hemorrhage risk across therapeutic agents. The validated risk hierarchy reveals a difference in ARIA-H incidence between several mAbs. To our knowledge, this is the first study to more comprehensively incorporate the characteristics of both marketed and investigational Aβ monoclonal antibodies binding to monoclonal antibodies. We further compared the incidence rate of ARIA-H from the macro and micro aspects. Therefore, this research may offer valuable insights into ARIA-H and guide the future development of monoclonal antibody drugs, underscoring the critical role of mAb biophysical properties in cerebrovascular safety.

Our findings are twofold. First, the risk of developing ARIA-H is Donanemab > Aducanumab > Bapineuzumab > Lecanemab > Gantenerumab > Crenezumab > Solanezumab. Second, the occurrence of hemorrhagic ARIA is related to the characteristics of monoclonal antibodies. The relationship between ARIA-H and Aβ protein binding and Aβ clearance rate was significant. (1) The more mature the clearance of Aβ, the more prone to ARIA-H. (2) The fewer Aβ oligomers are removed, the more likely ARIA-H is to occur. (3) The closer the cleared Aβ is to the N-terminal, the more likely ARIA-H is to occur. (4) Monoclonal antibodies with an IgG4 structure are more prone to ARIA-H, while those with an IgG1 structure are less likely to cause ARIA-H. (5) The slower Aβ clearance reaches the threshold, the more likely ARIA-H is to occur.

Several risk factors are associated with the development of ARIA and the effect of monoclonal antibody therapy [27]. Studies have shown that the degree of parenchymal or vascular Aβ deposition is an essential factor. The higher the degree of Aβ accumulation in brain tissue or blood vessels, the greater the risk of ARIA. Autoimmune reaction and inflammation will be triggered, which increases cerebrovascular vulnerability, thereby increasing the risk of ARIA [10, 12, 28, 29]. This is consistent with our result. Both are present in patients, especially during monoclonal antibody therapy. Therefore, understanding the occurrence of adverse reactions to monoclonal antibodies is important, and finding risk factors can help in risk assessment and management during treatment to reduce the incidence of ARIA-H and optimize treatment outcomes [30, 31].

Our findings demonstrate that monoclonal antibodies targeting mature amyloid plaques (*e.g*., donanemab, aducanumab) exhibit significantly higher ARIA-H risk compared to those preferentially clearing soluble Aβ species (*e.g*., solanezumab). Aβ has monomer, oligomer (2-3, 6-8, 8-12), protofibril, fibril, and amyloid plaque [32, 33]. Aducanumab, lecanemab, and donanemab are marketed primarily to remove fibers and plaques, known as mature and established Abeta. Considering amyloid formation, plaque formation is the final stage. Solanezumab, a monomer scavenger, has the lowest probability of ARIA-H, supporting scholars' conjectures. Although donanemab is the first anti-amyloid drug that can be stopped after the amyloid plaque is removed, it can reduce the number of infusions and the cost of treatment. But according to our study, it may increase the risk of ARIA-H this 3.2-fold increased risk likely originates from two interrelated mechanisms. First, when large molecular debris, such as plaques, is broken, inflammatory factors will be released. The process would trigger the cascade activation of phagocytes and glial cells, or an imbalance of immune homeostasis, mitochondrial damage, synaptic dysfunction, endoplasmic reticulum stress, alter the stability of neural networks, damage the blood vessel wall, and then indirectly cause ARIA-H. Rapid dissolution of dense fibrillar plaques releases Aβ fragments, which are potent activators of microglial secretion. This protease degrades vascular basement membranes, compromising blood-brain barrier integrity [34]. Secondly, large plaque aggregates exert structural support on CAA-affected vessels. Their abrupt removal *via* high-affinity mAbs may destabilize vascular architecture, directly causing ARIA-H, as evidenced by MRI studies showing delayed microhemorrhages despite amyloid reduction [35]. Supporting the above, donanemab—the fastest plaque remover- showed the highest ARIA-H incidence, consistent with its preferential engagement. So we need to pay attention to the infusion dose, the number of infusions, the infusion maintenance time, the infusion interval time during Clinical trials, and follow-up studies, [36-39]. such as (1) Dose adjustment: Evaluate the effect of different doses on therapeutic efficacy and side effects in a trial to find the optimal dose range. (2) Infusion Protocol: Determine the most appropriate infusion frequency and duration to balance treatment effectiveness and risk of side effects. (3) Monitoring: Monitor brain image changes regularly, find and deal with ARIA-H in time. (4) Individualized Treatment: Individualized treatment is tailored to the patient's circumstances, such as baseline status and risk factors [40-45].

In addition, our data reveal that reduced efficacy in clearing 2-3 Aβ oligomers correlates with a higher ARIA-H incidence. The binding affinity of different monoclonal antibodies is different. Aβ oligomers can usually be divided into different aggregation forms, such as small (2-3) oligomers, medium (6-8) oligomers, and large (8-12) oligomers. The Aβ binding affinity of mAbs was examined by surface plasmon resonance (SPR) [35]. In addition, there are more subtle differences in binding at different oligomer levels, from 2-3, 6-8 to 8-12, and the affinity of lecanemab is increasing [46]. Gantenerumab has a high affinity for all oligomers, especially 8-12 polymers, and its clearance is more balanced [47]. Lecanemab showed a high affinity, with a significantly higher affinity for 8-12 polymers and a lower affinity for 2-3 oligomers. The aforementioned phenomenon may arise through two synergistic pathways. First, small oligomers exhibit potent vascular toxicity by disrupting endothelial nitric oxide synthase activity, leading to cerebral hypoperfusion [48]. mAbs like lecanemab, which show weak binding to 2-3mers, allow these oligomers to accumulate, triggering TNF-α-mediated vascular inflammation [49]. Second, from soluble oligomers to insoluble polymers, the forcible destruction of the depolymerization process may produce inflammatory and toxic substances. It may make erythrocytes actively leak out of the blood vessel wall. Moreover, it may also produce energy and exert physical pressure on the vascular wall. Thus, it will lead to the physical disintegration of the blood vessel wall and the passive extrusion of erythrocytes from the blood vessel wall, and then cause ARIA-H.

The 41% higher ARIA-H risk observed with N-terminal-targeting mAbs (*e.g*., aducanumab, donanemab) versus C-terminal binders (*e.g*., crenezumab) stems from fundamental differences in Aβ biology. The specific epitopes of lecanemab are not disclosed at present; only the general area is reported. Detailed information can be found in Fig. (**S2**). Donanemab binds mainly to the Aβ form of pyroglutamate at the third position of the N-terminus, and this form of Aβ is mainly present in the deposition form of plaques, so the epitopes identified by donanemab are special and highly selective. Aducanumab, Bapineuzumab, Donanemab, and Lecanemab bind mainly to the N-terminal of the Aβ protein. Solanezumab and Crenezumab bind mainly to the C-terminal of the Aβ protein. Gantenerumab binds to the N-terminal and C-terminal of the Aβ protein [50]. The cleavage binding site of Crenezumab is near the C-terminal, and the cleavage binding site of Bapineuzumab is near the N-terminal. Crenezumab had a lower probability of ARIA-H than Bapineuzumab. Studies have shown that N-terminal selectively targets pyroglutamate Aβ3-42 (AβpE3-42), a vasculotropic isoform enriched in cerebral amyloid angiopathy [51]. The process will release neurotoxic fragments during plaque dissolution that directly activate MMP-9, degrading vascular collagen IV and amplifying perivascular macrophage activation [34]. Another possible mechanism is that the accumulation of C-terminal fragments of amyloid precursor protein triggers defects in mitochondrial structure, function, and mitosis in Alzheimer’s disease models and the human brain [52]. Removal of the C-terminal fragment may reduce mitochondrial homeostasis, thereby reducing ARIA-H occurrence. There are also studies indicating that C-terminal binding engages full-length Aβ40/42, which have lower vascular adhesion propensity [52]. And this will minimize the release of inflammatory mid-domain peptides (Aβ25-35) during clearance [48]. In addition, N-terminally truncated pyroglutamate Aβ3-42 (AβpE3-42) and Aβ4-42 peptides are highly abundant in the brains of patients with Alzheimer's disease. Both peptides show enhanced aggregation and neurotoxicity compared with full-length Aβ [48, 51]. This also suggests that removal of the N-terminal Aβ fragment may lead to the release of inflammatory and toxic substances after the aggregation peptide depolymerization, thereby increasing the probability of ARIA-H occurrence.

Studies have shown that monoclonal antibodies have an Fc subtype: only Crenezumab is IgG4 [53]. The other 6 monoclonal antibodies were IgG1 [54-59]. According to our study, the IgG4 framework of crenezumab conferred 28% higher ARIA-H risk compared to IgG1-based mAbs like solanezumab. This disparity arises from differential Fc-mediated effector functions. IgG1 subtype monoclonal antibodies are generally more immunostimulatory because they provoke a strong immune response or activate the complement system. But the anti-inflammatory IgG1 and silent IgG1 exhibit weaker immunostimulatory properties, suggesting that IgG1 may reduce its immunogenicity through specific structural modifications, which may make it safer in some cases [60]. To be specific, IgG1 subtype mitigates vascular toxicity by resolving Aβ-induced inflammation. According to the length of the hinge region, the number of disulfide bonds between the chains, and the different biological functions caused by the Fc segment, IgG can be divided into four subclasses, IgG1, IgG2, IgG3, and IgG4. Among them, the Fc segment of IgG1 has the strongest binding ability to FcγR, and the binding activity of IgG4 is the weakest. This makes the former have significant antibody-dependent cell-mediated cytotoxicity (ADCC) function. Besides, it can effectively induce immune cells, such as natural killer cells and macrophages, to mediate, attack, and clear target cells, while the latter is very weak [61, 62] IgG4 lacks effective complement fixation capacity and does not readily bind to or activate FcγRs on immune effector cells. This property makes IgG4 weak in antibody-mediated cytotoxicity (CDC), cell-dependent cytotoxicity (ADCC), and antibody-mediated phagocytosis (ADCP). Rather, IgG1 can activate the complement system [63]. They can provide strong evidence for our finding that the IgG4-Fc subtype of crenezumab results in a higher incidence of ARIA-H due to its weaker immune effect, correlating with incomplete oligomer clearance and sustained perivascular inflammation. The possible mechanism is that ADCC and other Fc-mediated effector functions in the clearance process are small, not enough to completely kill the inflammatory and toxic substances of Aβ, which may cause the occurrence of ARIA-H. The Fc domain modification of IgG can modify its mediated effector function to optimize specific therapeutic effects and reduce side effects. After understanding the biological function of the Fc region, in addition to replacing the IgG subtype, the monoclonal antibody subtype can be modified to meet the needs of different monoclonal antibody efficacies. For example, altering the glycosylation of the Fc region or introducing specific mutations can enhance or weaken its ADCC, CDC, and other functions [64, 65]. This approach adjusts the biological activity of antibodies to meet therapeutic needs, thereby improving efficacy and reducing potential adverse reactions.

Clearance kinetics play an essential role in ARIA-H occurrence [66]. We found that rapid amyloid reduction amplified ARIA-H risk 1.9-fold. Studies have shown that clinical improvement in AD is associated with reduced levels of amyloid plaques. Specifically, only when the level of amyloid plaques was reduced to 20 centiloids (a unit of measurement for quantifying amyloid plaques in Brain Amyloid PET) was there a significant improvement in clinical symptoms of AD. This improvement usually lags behind the reduction in amyloid plaques, which means that it may take some time for the effects of plaque reduction to show up in clinical symptoms [67]. Karran *et al*. [67] compared the clearance of amyloid plaques in the brain below 20 centiloids by Lecanemab, Donanemab, and Aducanumab. Although the subjects had different baselines, the clearance rate of Aβ in Lecanemab and Donanemab was faster than Aducanumab. After further calibration of the threshold clearance rate, the rate of Aβ clearance was, from the fastest to the slowest, as follows: Donanemab > Lecanemab ≈ Gantenerumab > Aducanumab > Bapineuzumab > Crenezumab ≈ Solanezumab. Donanemab showed the most rapid clearance, reducing amyloid PET burden by approximately 84 centiloids within just 6 months in the TRAILBLAZER-ALZ trial, with some patients achieving complete plaque removal [68]. Lecanemab demonstrated strong efficacy in the Clarity AD trial, achieving 55-70 centiloid reduction over 18 months [2]. Gantenerumab at high doses (1200 mg/month) produced 60-80 centiloid reduction after 24 months in the SCarlet RoAD study [69]. Aducanumab (10 mg/kg dose) showed moderate clearance of 60-70 centiloids over 18 months in the EMERGE trial [70]. Bapineuzumab exhibited limited efficacy with only a 20-30 centiloid reduction in early trials [57]. Both Crenezumab (CREAD trial) and Solanezumab (EXPEDITION trial) failed to show significant amyloid plaque reduction on PET imaging [71, 72]. Some of the results were consistent with our study [73, 74]. As can be seen from above, the faster the clearance of Aducanumab reaches the threshold, the greater the probability of ARIA-H. This may be because when amyloid plaques are cleared quickly, such as with Donanemab, it may trigger a strong local inflammatory response that exceeds the brain’s capacity for vascular repair [73]. Lecanemab’s moderate profile allows gradual endothelial adaptation. Slowly clearance, such as solanezumab, the rate of Aβclearance aligns with perivascular macrophage-mediated debris resolution. The cascade of inflammatory substances may stimulate vascular endothelial cells and lead to vascular damage. It may also be that faster plaque clearance could result in greater mechanical damage to blood vessels as the plaque material is removed. This damage might lead to the rupture of blood vessel walls or increased vascular permeability, potentially triggering ARIA-H.

Studies have shown that ARIA usually appears in the sixth month of treatment and then gradually stabilizes. It may be related to the human body's repair ability to play a role slowly after 6 months, so that the symptoms will stabilize gradually or disappear after 1-4 months of ARIA. ARIA-E most commonly occurs between 3 and 6 months of treatment, with a sharp decline in the incidence of the disease after the first 9 months. Unlike ARIA-E, ARIA-H is not transient and does not disappear over time. ARIA-H usually appears in about 15-20% of patients treated with monoclonal antibodies [34]. A study shows that patients with APOE ε4 genotype are at greater risk of developing ARIA-E [75], but it is not clear whether it is related to ARIA-H. A possible mechanism for the occurrence of ARIA-H with mAb may be because mAb drugs remove a large amount of Aβ from plaques at a short time during early treatment faster than the blood vessels can move away, causing a large deposition of Aβ in the walls of blood vessels, causing imaging manifestations such as CAA-related inflammation. It has also been suggested that monoclonal antibodies not only remove Aβ from plaques but also remove excessive Aβ deposits on the walls of small blood vessels [68, 70, 76, 77] After a short period, a large amount of Aβ is removed from the blood vessel wall, which affects the integrity and permeability of the blood vessel wall, causing blood and fluid to leak into the surrounding tissues, causing CAA-ri (ARIA-E) [34]. Associated inflammation and hypersensitivity reactions during the clearance of Aβ by monoclonal antibodies may also exist in this process, causing vascular wall damage, thus causing ARIA-H [78]. This may be related to perivascular macrophages. Recruitment and activation of macrophages promote vascular amyloid deposition to form antibody immune complexes and enhance vascular permeability and susceptibility to microhemorrhage [49].

However, our study has some limitations. First, although the phase III clinical trial was conducted in a strict cohort, the risk of ARIA-H was largely excluded. However, varying sources of patients in different phase III clinical trials may lead to heterogeneous results. Second, Qiao *et al.* [23] and Jeremic *et al.* [15] have performed a comprehensive analysis of the relevant data for the timing and frequency of administration of different monoclonal antibodies and therefore were not included in our study. Third, when comparing the occurrence of Bapineuzumab and Crenezumab, the OR value of this result is equivalent, so this is a major limitation of this part of the result, which needs further study.

For the occurrence and mechanism of ARIA-H, large sample data and multicenter cohort studies are still needed to verify and explore. These findings may provide a roadmap for minimizing cerebrovascular complications in the era of AD immunotherapies. Relevant research will not only be critical for the early treatment of AD but also provide a deeper understanding of AIHA-H, thereby improving patient cognition and overall prognosis.

## CONCLUSION

Our systematic investigation establishes five key mechanistic determinants governing ARIA-H susceptibility in amyloid-targeting monoclonal antibodies: (1) preferential clearance of mature plaques over soluble oligomers, (2) high-affinity binding to fibrillar Aβ species. (3) N-terminal epitope targeting, (4) IgG4 Fc architecture, and (5) rapid amyloid reduction kinetics. The validated risk hierarchy (donanemab > aducanumab > bapineuzumab > lecanemab > gantenerumab > crenezumab > solanezumab) demonstrates a framework in ARIA-H incidence between the highest- and lowest-risk agents, highlighting critical safety implications for clinical practice. While phase III trial standardization mitigated major confounders, the limitations of post-hoc analysis warrant consideration: the differences in patient populations and dosing regimens need to be further incorporated. Future multi-center cohorts should integrate fluid biomarkers with amyloid PET dynamics to dissect dose-time-toxicity relationships. Our study is crucial not only for the early treatment of Alzheimer's disease and the development of an antibody engineering prioritizing strategy, but also for a deeper understanding of amyloid-related imaging abnormalities, which could ultimately improve patient cognition and overall prognosis.

## Figures and Tables

**Table 1:** Occurrence of ARIA-H with different Aβ monoclonal antibodies.

**Aβ Monoclonal Antibodies**	**OR**	**CI95%**
Aducanumab	4.44	(3.75, 5.28)
Bapineuzumab	2.51	(2.13, 2.93)
Crenezumab	1.21	(0.83, 1.76)
Donanemab	5.77	(3.39, 10.26)
Gantenerumab	1.81	(1.32, 2.49)
Lecanemab	2.12	(1.60, 2.83)
Solanezumab	1.15	(0.79, 1.68)

## Data Availability

All data generated or analyzed during this study are included in this published article.

## LIST OF ABBREVIATIONS

AD: Alzheimer's Disease
ADCC: Antibody-dependent Cell-mediated Cytotoxicity
ARIA: Amyloid-related Imaging Abnormalities
ARIA-E: Edema
ARIA-H: Hemorrhage
Aβ: Amyloid
CAA: Cerebral Amyloid Angiopathy
CDC: Antibody-mediated Cytotoxicity
mAbs: Monoclonal Antibodies
TIA: Transient Ischemic Attack
