# Supplementary material for: Structural and Functional Determinants of ARIA-H Risk in Anti-Amyloid Monoclonal Antibodies: A Comparative Mechanistic Framework for Alzheimer's Immunotherapy Development
Source: Curr Neuropharmacol. 2025 Aug 19;24(7):1042–54. doi: 10.2174/011570159X391766250806091602 (PMC13523360; doi:10.2174/011570159X391766250806091602)
Supplement: Supplementary file 1 [file CN-24-7-1042_SD1.pdf]

Supplementary Material

Structural and Functional Determinants of ARIA-H Risk in Anti-Amyloid Monoclonal Antibodies: A Comparative Mechanistic Framework for Alzheimer's Immunotherapy Development

Dinghao An[1,2], Xinxin Zou[2,3,4] and Yun Xu[1,2,3,4,*]

[1]Department of Neurology, Nanjing Drum Tower Hospital, Chinese Academy of Medical Sciences & Peking Union Medical College, Nanjing, China; [2]Department of Neurology, Nanjing Drum Tower Hospital, Affiliated Hospital of Medical School, Nanjing University, Nanjing, China; [3]Jiangsu Key Laboratory for Molecular Medicine and Institute of Translational Medicine for Brain Critical Diseases, Nanjing University, Nanjing, China; [4]Nanjing Neurology Clinical Medical Center, and Nanjing Gulou Hospital Brain disease and brain Science Center, Nanjing, China

**Aβ monoclonal antibodies**	**Monomer**	**Oligomers**	**Protofibrils**	**Fibrils**	**Plaques**
Aducanumab	Weak	Yes	No	Yes	Yes
Bapineuzumab	Yes	Yes	No	Yes	No
Crenezumab	Yes	Yes	No	Yes	No
Donanemab	No	No	No	Yes	Yes
Gantenerumab	Weak	Yes	No	Yes	Yes
Lecanemab	Weak	Yes	Yes	Yes	Yes
Solanezumab	Yes	No	No	No	No

Fig. (S1). Binding of Aβ monoclonal antibodies. This image illustrates the ability of different monoclonal antibodies binding to different forms of amyloid. Yes represents binding, No represents almost no binding, and weak represents very little binding.

Aβ amino acid numbering	1	2	3	4	5	6	7	8	9	10	11	12	13	14	15	16	17	18	19	20	21	22	23	24	25	26	27	28	29	30	lgG class	Monomer/fibril preference
Amino acid	D	A	E	F	R	H	D	S	G	Y	E	V	H	H	Q	K	L	V	F	F	A	E	D	V	G	S	N	K	G	A		
Bapineuzumab	■	■	■	■	■	■																									lgG1	M=F
Lecanemab	Epitope undisclosed but between amino acids 1 and 16																														lgG1	M<<F
Gantenerumab	■	■	■	■	■	■	■	■	■	■	■												■	■	■						lgG1	M<<F
Aducanumab			■	■	■	■	■																								lgG1	M<<F
Donanemab			■	■	■	■	■	■	■																						lgG1	M=F
Solanezumab																■	■	■	■	■	■	■	■	■	■	■	■				lgG1	M>>>F
Crenezumab													■	■	■	■	■	■	■	■	■	■	■	■							LgG4	M>>>F
																														■	Key amino acid epitopes	

Fig. (S2). Aβ monoclonal antibody binding sites. Amino acid: A:Alanine: D:Aspantic acid. E:Glutamic acid: F:Phenylalanine, G:Glycine, H:Histidine, K:Lysine: L:Leucine. N:Asparagine:O:Glutamine: R:Arginine: S:Serine: V:Valine: Y:Tyrosine. M: monomer. F: fibril. This image illustrates the binding sites of different monoclonal antibodies, showing the amino acid sequence. Position 1 represents the N-terminal, while position 30 represents the C-terminal. The red markings indicate specific binding amino acid sequences within the epitopes.

1875-6190/26 $65.00+.00 **© 2026 Bentham Science Publishers**

Table S1. Occurrence of ARIA-H with different Aβ monoclonal antibodies (1).

ARIA-H, OR(95%CI)								
Aducanumab	Aducanumab							
Crenezumab	3.68(2.43,5.33)	Crenezumab						
Donanemab	0.77(0.42,1.35)	0.21(0.11,0.40)	Donanemab					
Gantenerumab	2.45(1.71,3.51)	0.67(0.41,1.09)	3.19(1.71,6.14)	Gantenerumab				
Lecanemab	2.10(1.50,2.92)	0.57(0.36,0.91)	2.72(1.49,5.17)	0.85(0.56,1.31)	Lecanemab			
Placebo	4.44(3.75,5.28)	1.21(0.83,1.76)	5.77(3.39,10.26)	1.81(1.32,2.49)	2.12(1.60,2.83)	Placebo		
Ponezumab	3.77(0.11,55.96)	1.02(0.03,15.52)	4.90(0.14,76.55)	1.54(0.04,23.12)	1.80(0.05,27.07)	0.85(0.02,12.54)	Ponezumab	
Solanezumab	5.09(3.37,7.73)	1.39(0.82,2.36)	6.63(3.45,13.20)	2.08(1.27,3.42)	2.43(1.52,3.92)	1.15(0.79,1.68)	1.35(0.09,48.16)	Solanezumab

Table S2. Occurrence of ARIA-H with different Aβ monoclonal antibodies (2).

Outcome	Drug(s) included in meta-analysis	Effect size(SMD or RR),(CI, 95% Confidence Interval)
ARIA-H(cerebral microhemorrhages)	Aducanumab	RR=2.69(CI=2.00;3.62)
	Bapineuzumab	RR=2.13(CI=1.40;3.22)
	Lecanemab	RR=1.81(CI=1.05;3.11)
